# Protracted hyperthermia and delayed rhabdomyolysis in ecstasy toxicity: A case report

**DOI:** 10.1097/MD.0000000000021842

**Published:** 2020-10-09

**Authors:** Arash Ghaffari-Rafi, Ki Suk Eum, Jesus Villanueva, Jay Jahanmir

**Affiliations:** a University of Hawaiʻi at Mānoa, John A. Burns School of Medicine, Honolulu, HI; b University College London, Queen Square Institute of Neurology, London, UK; c Tripler Army Medical Center, Department of Medicine, Honolulu, HI.

**Keywords:** carvedilol, ecstasy, hyperpyrexia, hyperthermia, MDMA, propofol, rhabdomyolysis

## Abstract

**Rationale:**

Despite toxicity and unpredictable adverse effects, ecstasy use has increased in the United States. Onset of hyperpyrexia, rhabdomyolysis, disseminated intravascular coagulation (DIC), among other symptoms, occurs within hours of ingestion. Moreover, patients who experience hyperpyrexia, altered mental status, DIC, and multiorgan failure, rarely survive. This case presents a chronic ecstasy user whose symptoms would have predicted mortality. The report demonstrates a patient who experiences protracted hyperthermia, with delayed rhabdomyolysis and DIC. In addition, his peak creatine kinase (CK) of 409,440 U/L was far greater than the expected 30,000 to 100,000 U/L, being the second largest CK recorded in a survivor.

**Patient concerns:**

This case report presents a 20-year-old man who presented to the emergency department after experiencing a severe reaction to ecstasy. He was a chronic user who took his baseline dosage while performing at a music event. He experienced hyperpyrexia immediately (106.5°F) while becoming stiff and unresponsive. Before emergency medical service arrival, his friends placed cold compresses on the patient and rested him in an ice filled bathtub.

**Diagnoses:**

Per history from patient's friends and toxicology results, the patient was diagnosed with ecstasy overdose, which evolved to include protracted hyperthermia and delayed rhabdomyolysis.

**Interventions:**

Due to a Glasgow coma scale score of 5, he was intubated and sedated with a propofol maintenance. Hyperpyrexia resolved (temperature dropped to 99.1°F) after start of propofol maintenance. He was extubated after 24 hours, upon which he experienced hyperthermia (101.4°F at 48 hours), delayed rhabdomyolysis, and DIC (onset at 37 hours). He remained in hyperthermia for 120 hours until carvedilol permanently returned his temperature to baseline. His plasma CK reached a peak of 409,440 U/L at 35 hours.

**Outcomes:**

After primary management with intravenous fluids, the patient returned to baseline health without any consequences and was discharged after 8 days. A follow-up of 3 months postdischarge revealed no complications or disability.

**Lessons:**

Clinically, the case highlights how physicians should be aware of the unusual time course adverse effects of ecstasy can have. Lastly, as intensity and duration of hyperpyrexia are predictors of mortality, our case indicates maintenance of sedation with propofol and use of oral carvedilol; both are efficacious for temperature reduction in ecstasy toxicity.

## 1 Introduction

A potent party drug, 3,4-methylenedioxymethamphetamine (MDMA) or ecstasy, has become more available in the United States (US), one of the most accessible drugs to teens and young adults.^[1]^ With the corresponding increase in MDMA use, US emergency departments (EDs) have observed a 58% rise in related visits between 1999 and 2000, with 80% of these visits being patients 25 years or younger.^[1]^ In Hawai’i, MDMA use is significantly higher, with a prevalence rate of 9.4% (2010–2011) for having ever used ecstasy, versus 6.4% in the entire US.^[2]^ Meanwhile, despite the increased use and life-threatening adverse effects, the percentage of young adults perceiving harmfulness of MDMA has hovered between 34% and 38% (from 1997 to 2000).^[1]^

MDMA has an array of symptoms. The minor includes diaphoresis, tachycardia, mydriasis, hypertension, xerostomia, bruxism, ataxia, nystagmus, confusion, and elevated mood.^[3]^ Rarer adverse effects include cerebral hemorrhage, cerebral venous sinus thrombosis, aplastic anemia, pneumothorax, and pneumomediastinum.^[3]^ Meanwhile, life-threatening symptoms include the syndrome of hyperpyrexia with rhabdomyolysis and multiorgan failure (HRMF), disseminated intravascular coagulation (DIC), serotonin syndrome, hyponatremia with cerebral edema, liver failure, and sudden death.^[3]^ In the face of MDMA-induced organ damage, serum creatine kinase (CK) rises to a maximum ranging from 30,000 to 100,000 U/L; however, the largest value recorded in a survivor was at 555,000 U/L.^[3,4]^ Paralleling the organ failure, patients generally experience prolonged hyperthermia (99.5°F–100.9°F) or hyperpyrexia (104.0°F–105.8°F), with higher peak temperatures associated with poorer survival outcomes; for MDMA-overdose, temperatures range from 104.0°F to 107.6°F.^[3,5–7]^ Overall, by impacting nearly all organ systems, MDMA toxicity requires complex medical management.

This case report presents a patient in extremis from polydrug overdose [MDMA, 3,4-methylenedioxyamphetamine (MDA), and marijuana] who makes a fully recovery, despite multiorgan failure. In addition, our patient exhibited a severe unexpected rhabdomyolysis and DIC, 38 hours delayed after initial ingestion of MDMA. The patient also exhibited the second largest recorded peak CK (409,440 U/L) in a survivor and largest peak for delayed rhabdomyolysis. We also uniquely report support for propofol and carvedilol in controlling chronically elevated temperatures in MDMA toxicity. This report highlights how temperature control is a critical element for managing patients with MDMA toxicity who otherwise have high mortality risk.

Most importantly, this case emphasizes an unusual time course of delayed rhabdomyolysis and DIC, along with protracted hyperthermia. Awareness that adverse effects can have onset or exacerbation many hours after initial MDMA consumption, when the patient has nearly returned to baseline health, can provide the managing team with foresight to remain weary for life-threatening complications when least expected.

## 2 Case

The patient is a 20-year-old Caucasian male who presented to the ED for altered mental status and hyperthermia secondary to MDMA and MDA use. He was playing the guitar at a music event where he took several doses of MDMA [which he suspects was mixed with benzylpiperazine ([BZP)] and MDA, for a total of 450 to 500 mg. The substances were powder in clear capsules consumed orally. After taking his first dose, the patient was noted to be running around bursting with energy, with mydriasis and bilateral nystagmus. Upon taking his second and final dose, the patient began rolling on the floor unresponsive and grunting. His face turned pale, lips became blue, and his skin was warm to touch. The patient's friends placed cold compresses on him, but the cold compresses quickly became warm. Subsequently, the patient became stiff, leading his friends to place him into a bathtub filled with cold water and ice. Eventually, the patient became agitated and angry, hitting and cursing at his girlfriend.

When emergency medical services (EMS) arrived, the patient had a Glasgow coma scale (GCS) score of 8, with dilated pupils, a temperature of 105°F, a heart rate in the 160 seconds, and a glucose of 46 mg/dL. He then hit a maximum temperature of 106.3°F and his GCS dropped to 5. He was intubated with 10 mg etomidate and 80 mg rocuronium, and per poison control, a propofol drip was used for maintenance. He initially received 4 L of normal saline, 2 L of lactated Ringer, 2 doses of 2 mg lorazepam, 1 ampule of 50% dextrose, 5 units of insulin, 1 ampule of bicarbonate, 1 g of calcium gluconate, and polystyrene sulfonate. Treatment lowered his potassium from 8.2 to 5.1 mEq/L. After stabilizing, a spontaneous breathing trial was conducted, and the patient was extubated within 24 hours from admission.

His past medical history was unremarkable, expect for chronic MDMA use. For the past 2 years, the patient would take MDMA once every 1 to 2 months, believing the substance was safe if his doses were spaced out in time. The patient noted to also smoke marijuana, but denied present or past use of alcohol, cocaine, heroin, methamphetamine, and any other substances.

Throughout his hospital course, the patient experienced rhabdomyolysis, DIC, and hyperpyrexia/hyperthermia. Regarding his rhabdomyolysis, on admission, his plasma CK was 38,659 U/L, peaking at 35 hours to 409,440 U/L, and only normalizing after 260 hours (10 days from admission). To prevent renal damage, his urine output was maintained at 200to 300 mL/hr via intravenous and per os (PO) fluids. Just after 24 hours from admission, the patient developed bilateral 5–7 cm posterior arm bruises and experienced swelling in all four extremities (deep venous thrombosis was ruled out). At 37 hours, the patient experienced DIC, with platelet count dropping to 22,000 platelets/μL and fibrinogen to 139 mg/dL, but recovering after 168 hours (7 days); platelets and fibrinogen did not drop below levels for transfusion. He also experienced signs of liver failure with elevated markers of liver dysfunction, which returned to baseline after 192 hours (8 days) from admission. The patient experienced hyperpyrexia and hyperthermia for roughly 168 hours (7 days). His peak temperature was at 106.3°F, which was managed with external cooling methods. During intubation and sedation with propofol, his temperature dropped to 99.1°F, but upon extubating, his temperature rose to 101.4°F. After 120 hours of hyperthermia, 3.125 mg bis in die (BID) PO carvedilol was initiated, which followed with his temperature declining to normal. Carvedilol was discontinued before discharge and he remained euthermic.

## 3 Discussion

### 3.1 Perceived harmfulness of MDMA

From US ED data, recreational use (2295 reports) was determined as the most common motive for consuming MDMA, followed by dependence (971 reports) and suicide (341 reports).^[1]^ As with our patient, many perceive MDMA as a safe substance for enhancing mood, with 38% to 49% of young adults (19–30 years old) in 2000 considering experimental MDMA use as dangerous and some doubting MDMA alone can kill.^[3,8,9]^ However, even in the purest forms, there are many life-threatening toxicities associated with MDMA; of note, as MDMA is purchased on the street, the substance's purity and dosage will be questionable.^[10,11]^ In addition to the difficulty in knowing the purity of MDMA tablets, the immediate effects can vary, with most ED visits due to unexpected reactions and overdose.^[1]^

### 3.2 Unpredictable effects

Although there are some universal effects (i.e., tachycardia, enhanced energy, empathy, and euphoria), there is difficulty in predicting the severe consequences, including sudden death, multi-organ failure, hyponatremia with cerebral edema, isolated liver failure, serotonin syndrome, rhabdomyolysis, and hyperpyrexia/hyperthermia.^[3]^ Several rationales account for the unpredictability of MDMA toxicity.

First, toxic reactions may depend on polydrug use and various adulterants in MDMA pills.^[12]^ In Australia, 59% of ecstasy deaths involved the patient consuming MDMA with at least another drug—the most common substances being ethanol, opioids, benzodiazepines, and methamphetamine.^[13]^ In a 6-year monitoring study, 39% of tablets were found to only have MDMA, 49% included MDMA with another substance, and 15% had another substance with no MDMA.^[11]^ Likewise, a 15-year monitoring study found 78% of tablets consisted only of MDMA-like substances, with the remaining 22% contaminated with BZP, ketamine, lidocaine, procaine, ephedrine, caffeine, and methamphetamine.^[14]^ Of the 78% MDMA-only pills, the dosages ranged from 1 to 225 mg per tablet.^[14]^ Our patient noted use of marijuana (THC) and possible contamination of BZP in his pills; his urine drug screen was positive for THC, benzodiazepines, and amphetamines (negative for cocaine and opiates).

The variability of response to ecstasy can also be accounted for by the enzymes that metabolize MDMA.^[12]^ MDMA breakdown forms 2 products, 3,4-dihydroxy metabolites and MDA.^[15]^ The first enzyme to breakdown MDMA and MDA is CYP2D6, which exhibits genetic polymorphism.^[16]^ In Caucasians (as our patient), 5% to 9% are deficient in CYP2D6, resulting in poor metabolism and increased toxicity despite lower drug doses.^[16,17]^ The poor metabolism is compounded by MDMA also being a potent CYP2D6 inhibitor.^[18]^

Afterwards, catechol-o-methyltransferase (COMT) breaks down the primary 3,4-dihydroxy MDMA metabolite into 3,4-dihydroxymethamphetamine (HMMA).^[19]^ Genetic polymorphism is also exhibited for COMT, with roughly 25% of Caucasians having low COMT activity.^[20]^ Meanwhile, HMMA itself stimulates vasopressin release, resulting in excessive water retention and hyponatremia.^[21,22]^ Hence, the low COMT activity in our Caucasian patient may have accounted for a reduced vasopressin secretion and lack of hyponatremia. Likewise, having a CYP2D6 and/or COMT polymorphism may have reduced MDMA metabolism and prolonged the toxic reaction in our patient—leading to delayed rhabdomyolysis, DIC, and prolonged hyperthermia.

The complexity of ecstasy response is compounded by the development of tolerance or reverse tolerance in chronic users.^[11]^ Due to decreased desirable effects with time, some users may increase MDMA doses or engage in polydrug abuse (mixing MDMA with other stimulants or hallucinogens) to enhance desired response.^[11,23]^ Our patient denied increasing his MDMA dosage, assuming such to be true, he may have experienced sensitization (reverse tolerance) from chronic use. With MDMA sensitization, despite consuming the same dosage, the serotonergic adverse effects escalate in intensity and duration, yet the locomotor activities only increase in intensity.^[24]^ MDMA increases central serotonin levels, directly triggering rhabdomyolysis and hyperthermia, independent of environmental elements (i.e., hyperactivity, dehydration, or warm ambience).^[25–27]^ Hence, reverse tolerance may have resulted in the delayed rhabdomyolysis and prolonged hyperthermia.

### 3.3 Delayed rhabdomyolysis

**Figure 1:**
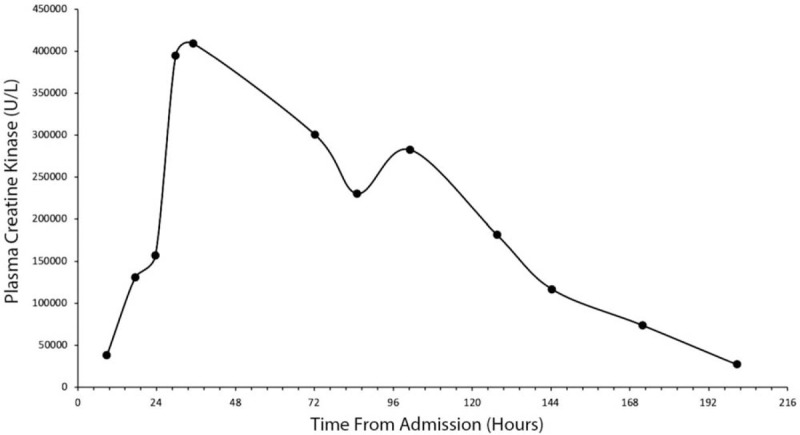
Trended plasma creatine kinase (CK): Plasma CK was trended from admission to discharge. Notably, CK levels reached a peak of 409,440 U/L at 35 hr from admission and 11 hr after extubating.

Generally, patients who present with hyperpyrexia, muscle stiffness, and hyperreflexia have rhabdomyolysis.^[3]^ Moreover, rhabdomyolysis is rapidly followed by impaired consciousness, DIC, and multiorgan failure, with few patients surviving this state (those who survive, do so after immediate treatment in the intensive care unit—as with our patient).^[3,28]^ Rhabdomyolysis often reaches a peak value between 30,000 to 100,000 U/L rapidly after MDMA ingestion.^[3]^ In our case, peak CK was reached 35 hours after hospitalization and 11 hours after the patient was extubated (Fig. 1). Only 2 other cases in the literature have reported delayed rhabdomyolysis: one after 30 hours from hospital admission, where CK peaked at 84,800 U/L; the second case was 55 hours after ingestion, with CK peaking at 116,032 U/L.^[29]^ In all cases, including ours, rhabdomyolysis was managed with vigorous intravenous and oral hydration.^[29,30]^ However, unlike the other cases of delayed rhabdomyolysis, our patient's CK peaked at 409,440 U/L becoming the second highest reported CK in a survivor (after 555,000 U/L), and highest recorded CK for delayed rhabdomyolysis.^[4]^ Notably, despite the 409,440 U/L CK value, at discharge, the patient returned to baseline health with no complications (including kidney damage).

### 3.4 Prolonged hyperthermia

**Figure 2:**
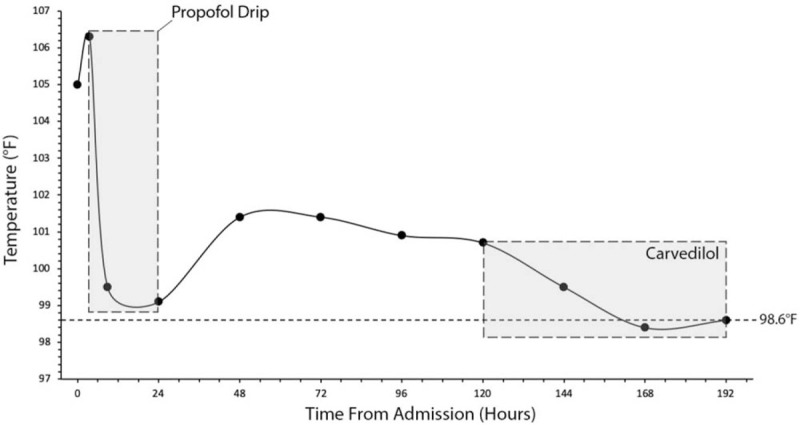
Trended body temperature. From ingestion until intubation, the patient exhibited hyperpyrexia. His temperature dropped during intubation with propofol maintenance for sedation, however rose quickly after extubating and discontinuing propofol. One hundred twenty hours from first EMS assessment, his temperature remained elevated until a dose of 3.125 mg carvedilol PO BID was provided. At discharge, carvedilol was discontinued at which point the temperature did not rise again. Time 0 hr represents first contact with EMS.

Maximum core temperature has been found to be associated with outcomes, with few survivors after 107.6°F; the maximum recorded in a survivor was 109.2°F.^[3,31]^ Hyperpyrexia's peak and duration is an indicator of the mortality and morbidity risk.^[3]^ From first contact with EMS, our patient remained in hyperpyrexia for 1 hour; his first MDMA ingestion was 2 hours before EMS contact (Fig. 2). The use of cold compresses and placement of the patient into an ice bath, by his friends, likely reduced the duration of hyperpyrexia and improved the patient's outcome. In the hospital, although external cooling methods were employed, the intubation and sedation maintenance with a propofol drip drastically reduced the patient's temperature from 106.3°F to 99.1°F (Fig. 2). Although only correlative, prior studies have demonstrated propofol to significantly reduce core body temperatures within 10 minutes of administration, by redistributing core heat to the periphery.^[32,33]^ Hence, when choosing an anesthetic for maintenance of sedation, our case supports utility of propofol for immediately controlling hyperpyrexia in MDMA toxicity.

All prior reported cases of MDMA use have indicated that temperatures normalize shortly after admission, with patients remaining euthermic afterwards.^[3,4,30]^ However, our patient experienced a rapid temperature elevation after extubation, subsequently remaining in hyperthermia for 168 hours. Only after administering carvedilol did hyperthermia resolve. Although our association in correlative, there is valid evidence from a randomized double-blind placebo-controlled trial on healthy humans, which supports use of 50 mg carvedilol for treating the hyperthermic and cardiovascular complications of MDMA use.^[34]^ MDMA's thermogenic effects result from stimulation of alpha-1 and beta-3 adrenoceptors.^[35,36]^ Although selective beta-adrenoceptor blockade is not recommended in stimulant users, due to risk of unopposed alpha-1 stimulation, beta-blockade was not found to affect blood pressure during MDMA use.^[37,38]^ Moreover, dual alpha and beta-blockade via labetalol or carvedilol was found to prevent cocaine's hemodynamic response without negative impact on cocaine-triggered coronary vasoconstriction.^[39–41]^ Hence, for MDMA toxicity, carvedilol should be standard of care for regulation of hyperthermia and cardiovascular complications.

## 4 Conclusion

Overall, our case highlights how clinicians should remain vigilant when treating MDMA toxicity, as the adverse effect can have onset or exacerbation hours after initial consumption. Knowing there is the potential for delayed rhabdomyolysis and DIC, as well as protracted hyperthermia, even if the patient has returned to baseline health, will provide foresight to management teams to be anticipatory of life-threatening complications when least expected. Moreover, this report supports utilization of carvedilol and propofol (for maintenance sedation) as a means for controlling hyperpyrexia/hyperthermia, the intensity and duration of which predict mortality risk. Lastly, we present a case where the peak CK rose to 409,440 U/L, being the second largest CK recorded in a survivor.

## Author contributions

All authors contributed equally to the development of the manuscript.

## Abbreviations

Abbreviations: BID = bis in die, BZP = benzylpiperazine, CK = creatine kinase, COMT = catechol-o-methyltransferase, DIC = disseminated intravascular coagulation, ED = emergency department, EMS = emergency medical services, GCS = Glasgow coma scale, HMMA = 3,4-dihydroxymethamphetamine, MDA = 3,4-methylenedioxyamphetamine, MDMA = 3,4-methylenedioxymethamphetamine, PO = per os, US = United States.
